# A Qualitative Study of Understanding Reasons for Self-Harm in Adolescent Girls

**DOI:** 10.3390/ijerph18073361

**Published:** 2021-03-24

**Authors:** Michelle Miller, Marcus Redley, Paul O. Wilkinson

**Affiliations:** 1 Department of Psychology, University of London, London EC1V 0HB, UK; michelle.miller.2@city.ac.uk; 2 School of Health Sciences, University of East Anglia, Norwich NR4 7TJ, UK; M.Redley@uea.ac.uk; 3 Department of Psychiatry, University of Cambridge, Cambridge CB2 0SZ, UK

**Keywords:** self-harm, NSSI, adolescent, compulsivity, impulsivity, interpersonal

## Abstract

*Objective:* Self-harm is an important public health issue in the UK. Young people who self-harm frequently feel misunderstood, and unable to access help. Improving understanding is key to informing the development and delivery of effective treatments and services. *Methods:* In this qualitative study, we interviewed nine adolescent girls (13–17 years old) with recurrent self-harm, recruited from NHS specialist child and adolescent mental health services. Data were analysed using Interpretative Phenomenological Analysis (IPA). *Results:* Findings revealed that self-harm is experienced as powerful mental and physical urges, sated only by self-harming, suggesting that self-harm could be considered a compulsive rather than impulsive disorder, representing a new perspective on the behaviour. Five themes emerged: emotion regulation; an addictive urge; self-harm to survive; interpersonal triggers; interpersonal relationships, not mechanical distractors, reduce self-harm. *Conclusions:* This study provides further evidence that non-suicidal self-injury may be engaged in to reduce suicidal risk. Seeking the company of helpful friends or family members may reduce the urge to self-harm. Repetitive self-harm may be a compulsive behaviour.

- *Self-harm is preceded by powerful mental and physical urges, sated only by self-harming.*
- *Self-harm could be considered a compulsive rather than impulsive disorder, representing a new perspective on this behaviour.*
- *We need to incorporate an interpersonal perspective into intervention strategies.*

## 1. Introduction

Prevalence of self-harm is high, estimated at 17% in adolescents in a recent meta-analysis [1]. Reduction in self-harm is a key public health target in the UK and internationally [2]. While some self-harm is attempted suicide, there is increasing interest in non-suicidal self-injury (NSSI)—deliberately harming one’s body without any intent to end life. This delineation between suicidal and non-suicidal self-harm is controversial, with some considering the distinction valid but others arguing that suicidal and non-suicidal self-harm should not be separated, given that intent is often unclear, and many people engage in both [3,4]. Authors of questionnaire studies have identified reasons for NSSI including reducing unpleasant affect and communicating distress [5,6]. However, such quantitative studies cannot provide a deeper understanding of these motives nor demonstrate reasons that are not options on the questionnaires. To provide optimal treatment, we need to understand the motives in depth.

The National Institute for Clinical Excellence [7] recommended using qualitative methods to explore the meaning and intentionality of self-harm. Despite this, few qualitative authors have interviewed people about their experiences of self-harm. Such studies provide more in-depth explanations of motives described in quantitative studies, including relief from distressing affect [8,9,10], partly because physical pain is easier to bear than emotional pain [8,11]; and self-punishment [8,9]. Rather than considering NSSI indistinguishable from suicide attempts, these authors have demonstrated that people use NSSI to reduce suicidal risk, due to the reduction in distress [11,12]. Such research has also shown the concept of NSSI to be useful, by demonstrating a subjective distinction between suicidal and non-suicidal self-harm [8,11] and by demonstrating that professionals’ assumption that patients are suicidal is unhelpful [8].

Adolescence is the peak age for both prevalence and new incidences of self-harm [13,14]. However, all but two [10,15] qualitative studies have focused on young adults, with one focused solely on young people in care [10].

The literature suggests overlapping, but differing, motivations for self-harm between boys and girls. Girls tend to engage in self-harm for intrapersonal reasons more than boys: ‘to communicate distress’, ‘to quell distressing thoughts’ or to ‘relieve tension’ [16,17], whereas males are more likely to report reasons implying a desire to ‘frighten someone’ or ‘to die’ [6,18]. Literature suggests that males and females are socialised to process their emotions differently, for example, anger versus shame, and therefore manage these emotions in different ways [19]. Females tend to ruminate and internalise their problems more than males, which may lead to more self-harm for intrapersonal reasons [19]. Sornberger et al. (2012) [20] found that females tend to use methods that result in bleeding; for some people, seeing blood is an important aspect of NSSI, a feature identified in this study. These potential gender differences suggest that it would not be appropriate to ignore gender of participants in a qualitative study of self-harm. Such a study would need to either have significant numbers of participants of each gender, so that similarities and differences could be explored; or choose one gender only and explicitly state the limitation that results should be transferable to that gender but may not apply to the other gender. Due to the practical limitations of conducting this student study, we chose the latter approach: to deepen understanding of the experience of female adolescent self-harm.

### Rationale for a qualitative approach and Interpretative Phenomenological Analysis as a methodological choice.

The primary purpose of this study was to deepen understanding of the lived experience of female adolescent self-harm. In particular, we hoped to explore the participants’ sense-making of their reasons to self-harm on some occasions and not to on others. Insight into the drivers to self-harm is equally important to understanding reasons not to engage in self harm, when one otherwise would. This information could offer new knowledge to the field and deepen our understanding of this complex phenomenon. In turn this could contribute to the development of treatment interventions.

An evidence base exists of the epidemiological factors underlying self-harm, established via numerous quantitative studies conducted in this field. While quantitative research has formed the foundation of evidence-based healthcare, quantitative methods are not intended to direct healthcare professionals to the essence of the patient’s subjective experience [21].

Qualitative research can facilitate an understanding of the complexity of phenomena, thus informing clinical practice (Boyle, 1991, cited in [21]) We suggest that paying attention to the lived experiences of self-harm could provide insights which contribute to the body of literature, help us to understand why people do (and do not) self-harm and further inform clinical intervention. These factors are implicated in our choice to adopt a qualitative approach to research and in selecting Interpretative Phenomenological Analysis (IPA) as our methodological choice, to gain a deeper understanding of participants’ lived experience of self-harm and to explore their sense-making of decisions to self-harm and not to self-harm, when they typically would. Interpretative phenomenological analysis (IPA) is a qualitative approach to research, which aims to examine in detail personal lived experience. IPA is phenomenological in its concern with the detailed examination of human lived experience and how individuals make sense of their experience [22]. A key feature of IPA investigation is the way in way in which it tries to enable the participant to express their experience in their own terms, instead of applying predefined and fixed methods. Within IPA, understanding people’s experiences and their relationships to the world is an interpretative endeavour, where we focus on participants’ attempts to make meanings out of their experiences, by means of hermeneutic interpretation. IPA subscribes to the idiographic view that phenomenological enquiry is an interpretative process, separating it from a purely narrative description of an experience. Interpretation is the means to uncovering, not simply describing phenomena [23]. This means that the researcher is able to offer a perspective, possibly unknown to the participant, manifesting that which may lie hidden [23]. This aspect emanates from Heidegger’s notion of ‘appearing,’ which requires the researcher to engage in detective uncovering of the phenomenon to bring it forth and make sense of it [22] (p. 35). The researcher engages in critical analysis of the data, ‘in ways in which the participant might be unwilling or unable to do themselves’ [24] (p. 189). Interpretation is a cyclical process, whereby the researcher is required to be reflective throughout the process, to acknowledge the appearance of preconceptions, as they arrive and infiltrate thinking. This dynamic interpretative interplay between the ‘whole’—the researcher’s ongoing biography, and the ‘part’—the encounter with the participant, represents the hermeneutic circle [22]. Within this encounter, the researcher attempts to ask questions not only of the data, but also of her response to the encounter and the data derived therefrom [22].

Smith & Osborn (2003) added another dimension to this hermeneutic sense-making process; they formulated the idea that IPA involves a double hermeneutic, reflected in the dual role of the researcher and the position of the participant in interpreting the experience: the researcher is making sense of the participant, who is making sense of their experience [22]. Researcher access to the participant’s experience is limited; the aim is to get ‘experience close’ and move away from a direct description of the experience to provide an illuminating interpretation, through a deeper analysis, that is no less grounded in the interview data (Conrad, 1987, cited in [22]). IPA utilises both phenomenological and hermeneutic insights in its endeavour to uncover the hidden phenomenon, from within, rather than importing a theoretical perspective from outside the data [23]. IPA does not eschew the use of external theory but connects it to findings in order to show how the case may relate to other relevant research [23]. Therefore, IPA produces an account of lived experience in its own terms rather than imposing prescribed pre-existing theoretical preconceptions. IPA is idiographic in its commitment to examining the detailed experience of individual cases, prior to moving to more general claims [25]. We utilised semi-structured interviews as a flexible data generating method, affording young people control and a sense of power within the interview process. This approach enabled us to generate non-numerical data, from which we gained an idiographic understanding of our participants’ experiences.

‘IPA is a particularly useful methodology for examining topics that are complex, ambiguous and emotionally laden’ [24] (p. 1). Self-harm is a prime exemplar of such a phenomenon: multifaceted, involving complex emotional and somatic interactions with self and others. Smith (2004) [26] suggests that IPA’s ‘idiographic commitment to the case’ supports researching individual accounts of mental and physical conditions. This is in line with the aims of the National Health Service to acknowledge the voices of service users and is in keeping with the increase in patient-centred research, which offers the possibility of new insights into health and illness [7,27,28].

Research Questions

1. How does an adolescent female make sense of her experience of self-harm?
2. How does an adolescent female make sense of her choice to self-harm on some occasions, while choosing not to on others, when she otherwise might?

## 2. Methods

### 2.1. Participants

Nine adolescents were recruited (via clinicians) from NHS child and adolescent mental health services (CAMHS) in Cambridge (five from an adolescent in-patient unit, four from a community clinic).

Participants were aged 13–17, female, with at least one act of self-harm in the past six months (we specifically decided to include all type of self-harm in choosing participants, allowing the analysis to consider motivation), current Cambridge NHS CAMHS patients, able to speak English well enough to give informed consent and take part in the interview. Diagnosis and suicidal risk did not affect inclusion/exclusion. Median age was 15 (range 14–17). Primary diagnoses assigned by consultant psychiatrists were unipolar depression [5], borderline personality disorder [2], unipolar depression/PTSD [1] and unipolar depression/panic disorder with agoraphobia [1]. Pseudonyms are used.

We recruited an all-girl sample for two reasons: due to potential differences in motivations between girls and boys (boys’ motivation leans more towards anger at self or others, while girls’ motivations are more likely to be ’self-punishment’ and ‘wanting to communicate distress’ [6,18]) and because IPA requires in-depth exploration of small homogenous sample groups [22]. This means that if we recruited boys as well, we would have needed a much larger sample to be able to establish similarities and differences across genders. This was not feasible in the scope of this project. While patients of clinical services do not represent the full population of young people who self-harm, we restricted the sample to patients of clinical services. This was for ethical reasons: the interviewer was a non-clinician student, and it was important to ensure good aftercare for participants who revealed self-harm at interview. (If we had recruited from the community, participants, who would all have revealed self-harm, would have needed review by a clinician according to local ethics rules; this itself would have made the sample non-representative.) Each participant had an allocated trained clinician who was able to speak with the patient about any distress resulting from the interview, to assess and manage risk, as appropriate. We accept that there may be some differences between the use of self-harm between young people in the community and when in-patients. However, in most cases, in-patients spoke about their use of self-harm when in the community. Additionally, while there are sometimes differences in severity of illness between British young people in hospital and in the community, there is great overlap and the main driver for admission is social support rather than illness severity.

We conducted a Patient Public Involvement (PPI) group with adolescents with lived experience of self-harm, prior to embarking on this study. The PPI participants reviewed the study design, questions and protocol and offered valuable feedback. The participants appreciated the idea of this study asking young people to voice their own experiences. They expressed a view that young people are frequently asked to participate in research but are rarely asked to share their experiences in their own words.

Young people aged 16 and over gave consent; those under 16 gave assent and parents provided consent. The study was approved by the National Research Ethics Service Essex Committee and Cambridgeshire and Peterborough NHS Foundation Trust R & D department.

### 2.2. Interviews and Context

To ensure the safety of vulnerable participants we conducted the study within settings that provided ongoing support. Conducting the interviews within in-patient and community clinics ensured that participants would be familiar with their surroundings and clinical staff and if necessary, could receive clinical support after an interview. The settings offered a twofold structure to the study: (a) adherence to ethical guidelines while ensuring the safety and support of the participants and (b) to provide support to the researcher.

Semi-structured interviews took place in the in-patient unit or community clinic from July to December 2015. The interviewer (MM) had no previous relationship with the young people and met with them alone. The interview guide was developed by the research team in collaboration with a PPI group of young people with lived experience of self-harm. Questions were opened ended, for example, “What can you tell me about your first experience of self-harming?” The interview guide is in the Supplementary Materials.

### 2.3. Ethical Considerations

This study was granted approval by (a) University of Cambridge, (b) NHS Trust (CPFT) Department of Research and Development and (c) Health Research Authority Local Research Ethics Committee East of England. The purpose of such regulation is to ensure that the advantages to society of the research outweigh the disadvantages; in particular that the rights of the participants prevail over the interests of research, that the study is ethically and safely conducted and that participants are able to consent. Written informed consent was obtained from a parent for participants under 16, alongside assent from the individual. Those aged 16 to 17 provided consent. The key ethical issues were: if a participant was revealed to have self-harmed recently and/or to be at significant suicide risk, it was essential clinical staff were informed of this so in a timely fashion so as to reduce risks to the participant (hence recruitment from NHS clinical services); true informed consent of the participant/parent (with a full participant information sheet, written with guidance from young people, and a delay between being informed about the study and the interview); and data security (following University of Cambridge data governance rules)

### 2.4. Interview Risk Protocol

We considered the potential risk for a participant to become distressed during the interview or to self-harm following the interview. We implemented a risk assessment strategy: the researcher assessed risk at the beginning and end of each interview and debriefed with her clinical supervisor after each interview, so that appropriate clinical action could be taken if necessary. The researcher monitored for signs of distress throughout interviews, allowed for breaks and sensitively approached the participant with questions, ensuring the participant was aware of her rights to stop the interview at any stage or refrain from answering a question.

### 2.5. Interview Protocol

The researcher introduced herself and explained her reasons for embarking on the study. The participant was invited to ask questions. Participants were verbally asked whether they consented before being invited to sign the consent form. The researcher explained that they were free to say as much as they felt comfortable and could end the interview at any time. The researcher demonstrated how the recorder worked and set it on a table, allowing participant control over the recorder.

The interviews were semi-structured. The researcher followed the participant’s lead as much as possible, asking prompt questions or ones relevant to their context. The interview could have taken 60 min as a maximum. The researcher remained mindful of the nature of their distress and erred on the side of caution, sensing when the participant seemed to draw the interview to a natural end.

### 2.6. Analysis

The data were analyzed using IPA to explore and gain insight into participants’ subjective experiences of self-harm. Individuals were invited to reflect on their self-harm and seek meaning in their embodied, cognitive-affective and existential domains [29,30]. We aimed to gain a deeper understanding of what self-harm meant to each participant. IPA is idiographic in nature, aiming to gain detailed understanding of first-person experiences, in particular contexts exploring each case, before moving to general statements [31], which were borne out in our finding of compulsivity, associated with repetitive self-harm, which was grounded in participants’ experiences.

Interviews were read and re-read before coding and analysis. This process led to emerging themes that were grounded in the data, which appeared both similarly and differently across cases. Emerging themes were grouped into identifiable consolidated themes.

### 2.7. Analytic Process

A process was followed from transcription to data analysis, according to the Smith et al. (2009) suggested IPA method. Interviews were recorded and transcribed; upon transcription, data were anonymised, pseudonyms were allocated to each participant and all personally identifiable information removed. Working case-by-case, the interviews were read and re-read. Initial thoughts and reflections were noted on the printed transcripts. The next stage was one of ‘exploratory coding’, which comprised reviewing the transcript, line by line, exploring three areas for interpretation: (a) Descriptive: focusing on content, (b) Linguistic: focusing on use of language, use of pronoun and pauses and (c) Conceptual: this required the researcher to engage at a more interrogative level, asking: ‘what could this mean?’ This process gave way to emerging themes that appeared both similarly and differently across cases.

Emerging themes, specific to each individual, were listed chronologically, in a table, with corresponding extracts from the transcript to ensure we had remained grounded in the data. These themes were grouped into identifiable Consolidated Themes, which were subsumed into Superordinate Themes, with the consolidated themes subsequently listed as sub-themes. The Superordinate Themes are Emotion Regulation: making internal feelings visible and concrete; An Addictive Urge: a Powerful Force that cannot be controlled; Self-Harm to live: a battle to survive; Interpersonal: triggers and regulators. Superordinate themes are themes that emerge from the data which seem most ‘potent’. They are particular to each individual and yet, when looking across the cases, they appear to represent ‘instances of higher order concepts’ [22] (p. 101). That is, they tell a unique story about ‘this particular person’ but also reflect more existential issues, applicable to a wider population. Attached to each superordinate theme are two to three sub themes, which illustrate the unique ways each individual exemplified each superordinate theme. These provide textural renditions to the superordinate themes and assist in their definition. Superordinate themes, supported by sub themes were linked to transcript extracts, which supported and illustrated each subordinate theme. The next stage is interpretation, which, in IPA aims to uncover the hidden phenomenon behind the manner in which it appears, and in so doing, the researcher may offer a perspective perhaps unknown to the participant [23]. We posed two research questions which explored how the person made sense of their self-harming and occasions when they did not self-harm. We subsequently selected transcript extracts, grounded in the data, which reflect the unique and phenomenological aspects of the analysis, and which answered our research questions, now presented in this study. In the discussion, we discussed our interpretations of the data and put them in the context of extant literature.

### 2.8. The Role of the Researcher in IPA

IPA acknowledges the central role of the researcher in influencing and constructing the research process and recognizes that a researcher’s presuppositions can both hinder and enhance interpretations of another’s lived experience as she/he attempts to explore the meanings and experiences of participants; the researcher’s own preconceptions and assumptions are implicated in the analytic process and have a bearing on the interpretation of the data [22,29,32].

## 3. Results

We posed two research questions: one, to investigate the experience of self-harm and two, to consider whether there were times when a participant did not self-harm and if so, what the contributing factors might be.

1. How does an adolescent female make sense of her experience of self-harm?
2. How does an adolescent female make sense of her choice to self-harm on some occasions, while choosing not to on others, when she otherwise might?

Based upon this question and grounded in the data, we identified the ‘experience of self-harm’ to encompass mental, emotional, physical and social features.

- Self-harm was expressed as an interpersonal means to express and communicate mental distress, to make internal pain visible to self and others, or to quell mental anguish by focusing instead on a physical pain; for example, the pain of cutting and watching the blood flow enabled a participant ‘to feel’—which was preferable to a state of ‘nothingness’. Intrapersonal features were identified in self-punishment and self-loathing.
- Self-harm was experienced as compulsive urges, within a ‘vicious cycle’ and as an internal battle between uncontrollable intrusive physical and mental urges, which were sated only by self-harming.
- All participants experienced a beneficial reduction in their urges to self-harm in the company of people who understood, tolerated or accepted them. The participants sought the company of family, friends or another, who, by their presence or soothing words, were able to calm the individuals and distract them from their need to self-harm. We suggest that this could be understood within the theoretical context of regulatory interpersonal relationships.

Five key themes emerged from the data. These are summarized below.

### 3.1. Theme 1: Emotion Regulation: Making Internal Feelings Visible and Concrete

Most participants in this study experienced tension preceding their self-harm and relief afterwards. The primary purpose of self-harm is regulation of affect–reducing intense distress; described by participants as: “distraction from suicide”, “externalise internal pain”, “to feel”, “release anger”, “numb distress of rape”. Self-harm does this partly by distracting from emotional pain.

*It takes my focus away from another kind of pain, like the pain inside, like the way I feel. So, it overtakes that so I kind of forget about the other sort of pain. (Lily)*

Lily’s experience exemplifies the theme of ‘making internal pain, externally visible’. She spoke frequently about ‘pain’, both emotional and in her body. The interviewer sensed her strong urge to express this internal pain, to communicate her internal state and alert others to how she was feeling. Self-harm was her call to help, when she felt that she had no other means to express herself or make it visible: ‘you can’t see it’. Finally, a hospital admission ensured this pain was now ‘out there’ and ‘understood’, thus enlisting the help she needed.

*You just feel relaxed, like all the feelings of pain, numbness, anger, they just go for a while—literally, they just go until my arm stops bleeding and then I feel like I need to do more. (Zoe)*

Participants described how physical pain led to a release of mental pain.

*I just felt that by cutting myself I was getting a release from all the anger, the guilt, the stress of everything that was going on. (Zoe)*

*It’s when it’s first done and first watching it and watching everything happen and unfold, is where you get that sensory feeling, of release and relief. (Davina)*

Relief. *It’s like a big weight has been lifted off my shoulders, I release today’s pain and I am ready for tomorrow. (Isla)*

Some young people described self-harming because they feel nothing yet want to feel something.

*I am doing it to feel pain to remind myself that I can still feel pain, because at the moment I feel nothing I feel numb and it’s my only way that I am reminding myself that I am still here, that I am still alive…by seeing the blood. (Isla)*

*And then there’s other times when I want to feel something and I don’t want to believe that I’m not feeling anything… so sometimes I want to know, I want to FEEL something. (Davina)*

Watching the blood drip from a cut provided some participants with a visceral dimension to their experience of emotional “release”. Sometimes they needed to see this blood to prove that they are still alive.

*I get this sense of release and I just love watching the blood drip from it, it makes me feel good. (Davina)*

*Seeing blood calms, me down quite a bit, as it comes out and it sort of drips. (Sophie)*

*There used to be a point where I used to self-harm to see the blood and to see the blood was like, ‘yea, you are still alive’. (Sophie)*

### 3.2. Theme 2: An Addictive Urge: A Powerful Force That Cannot Be Controlled

One of the most striking features found to the lived experience of self-harm resided in the nature of the urges. These powerful physical and mental forces urged the person to comply and were hard to resist. They were experienced as an urge that could only be sated by the act of self-harming. The participants seemed to battle these addictive, strong urges, at times for lengthy periods, while either trying to resist them or planning relief from them; suggestive of a compulsive nature to the urges, rather than the typically understood ‘impulsive’ urges.

Participants described strong cravings to self-harm:

“My body knows”; I have that craving, it’s like it will not stop until like, I hurt myself. (Lily)

It’s addicting, so at first you do it because you are really sad and you do it because there is so much inside of you, and you need to let it go and each cut would let a bit of emotion go and it has become a part of my life. I guess I can’t go without it, like I crave it. (Sophie)

These mental and physical urges were hard to control and only sated by self-harming.

*They (urges) just take over; you feel that you have no control left…It’s physical and mental, you feel that you have no control…progressively over the years it got worse and completely out of control. (Zoe)*

*It’s much more powerful than you, if it wasn’t then you wouldn’t want to self-harm. (Zoe)*

*One of my family members was saying that I must not want to get better and how it’s my fault that I am doing this and that I am in charge, but for me I am not in charge, I can’t control what I do. (Hannah)*

Self-harm can sometimes be delayed, but the urge must be acted on eventually.

*Self-harm is always sitting in your mind and if I am watching the telly or something, I’ll be thinking about it and it will be like, ‘Oh I need to self-harm, I didn’t self-harm earlier, I have to self-harm later.’ Then I build all these plans in my head for when I am going to self-harm, what I’m gonna use, what I’m gonna do after I self-harm, how I’m gonna deal with it. (Hananh)*

*“If it’s been a bad day, then late at night when everybody is asleep or I am on my own, in my room, then I do it…I mean if I am at school, I don’t self-harm in school.” (Charlotte)*

*“The more you wait, and then eventually when you do get to self-harm you let it all out.” (Lily)*

As self-harm became embedded, its impact reduced, and tolerance increased. This could lead to more damaging methods of self-harm.

*Because the small scratches and cuts that I was doing before don’t give me the release. I felt that I could cut deep and still have control over it and I did for a while, now I have no control over it. (Zoe)*

*I was 8 years old, I started head banging, then that continued to scratching and picking my skin and then I first started cutting when I was 11. (Paula)*

### 3.3. Theme 3: Self-Harm to Live—A Battle to Survive

In addition to being non-suicidal, NSSI can be experienced as a ‘means to survive, to not die’. Participants had intense negative affect, which led to suicidal thoughts. Part of them did not want to die, so they self-harmed to reduce the suicidal thoughts—and, thus, to live.

One participant had tried to cope with mounting academic pressure, bullying and stigma related to her self-harm, which led to suicide ideation; however, she did not want to die, she wanted the mental anguish to end. This is illustrated in her self-description: *“I’m a suicidal teenager and self-harm is MY way of LIVING”; “There’s a lot of stigma about self-harm and people feel that self-harm is a way of dying when it’s not, like self-harm for me and for a lot of other young people is we self-harm to LIVE, not to die.” (Hannah).*

*Some people do not understand, and some people will interpret self-harm as ‘just’ a way to die, but it’s not a way to die, it’s a way to keep living. (Sophie)*. This depicts how one participant experiences self-harm as her ‘fight to stay alive’, when completing suicide and dying, would be an easier option. ‘Living’ is a battle, which requires strength, hence the view that self-harm is utilised reduce suicidal risk.

*I think it’s a way of taking care of yourself, because I feel in a way like self-harm stops you thinking about suicide as well. (Sophie)*

### 3.4. Theme 4: Interpersonal Relationships as Triggers to Self-Harming

Problems in interpersonal relationships often triggered self-harm. Bullying at school and relationships with parents featured across the cases.

*I was bullied really, really badly, by my best friend and… that’s like kind of when I first started self-harming. (Isla)*

*It’s like at school they told me they were putting me down a few sets in maths…and then that night I went to my room and I was so frustrated, and I was so scared to tell my parents and I ended up self-harming. (Hannah)*

*“When I first started self-harming, I didn’t have a very good relationship with my parents, actually, our relationship was really bad, and I felt responsible for that.”(Charlotte)*

*“Every time, even now, if we (mother) try to talk about it we end up arguing and it escalates, and I usually end up self-harming during the nights.” (Lily)*

*“My dad’s way of dealing with things is pretending they are not happening; me and my dad have a very bad relationship.” (Paula)*

Sometimes criticism or teasing about the self-harm itself led to more self-harm.

*There’s all this stigma against self-harm and there’s people in your school that just won’t understand and will take the mickey out of you. Like I had a girl shouting out: ‘oh cutty, cutty, go cut’ and that would make me feel a million times worse and I would just go and self-harm again cos I felt awful. (Hannah)*

School pressures and bullying from peers triggered self-harm.

*“12 year olds, they’re children, they are trying to cope with everything….they just have too much to deal with because they are giving themselves too much pressure.” (Hannah)*

*“I remember, we would go out and we would have a laugh and we would just buy sweets and go on the park or something, but now it’s not like that at all, there is real pressure by their parents for exams.” (Hannah)*

*“I would quite often be bullied because I was a bit weird because like I didn’t really hang out with the girls very much, cos I identified more with the guys and they didn’t want me because ‘oh it’s a girl, we don’t want a girl’.” (Paula)*

### 3.5. Theme 5: Interpersonal Relationships, Not Mechanical Distractors Can Reduce Self-Harm

Many participants wanted to stop self-harming. Sometimes “mechanical” distraction methods such as wearing elastic bands or holding ice cubes had been recommended as alternatives to self-harm. However, participants did not find these methods effective.

*I pretty much tried everything; the elastic band, the drawing, tried everything, but it doesn’t give off the same effect. (Niahm)*

*I mean I’ve tried everything—there’s the elastic band on the wrist, you ping it when you feel like you wanna self-harm, there’s going and holding ice because it releases the same hormones as cutting does. (Hannah)*

*I tried all sorts of other coping mechanisms and nothing worked as well as self-harming did. (Paula)*

Hannah sought the company of others who might understand what she could be experiencing. In so doing, Hannah did not self-harm, when she otherwise might have. It appears that the company of others—who understood her and did not judge her, acted as a regulating factor for her and at those times, Hannah was offered an ‘alternative’ to self-harming. It appeared that people, rather than ‘mechanical methods’ were more beneficial to Hannah. As Hannah recalled these ‘mechanical methods’ she spoke as though the methods were of ‘academic’ but not practical interest.

Spending time with supportive people helped reduce the urge to self-harm.

*I don’t always give in, I turn to something else, like especially my mum, she’s like one of my biggest supporters in my life. We’re very, very close…I’ll go and sit with her and talk to her and it slowly goes away… So I don’t feel that way anymore. I might still feel like alone and upset and however, but I don’t have that urge to hurt myself. (Isla)*

Isla sought her mother’s support when she felt an urge to harm herself, in the belief that her mother would and could help her: Isla was calmed and soothed by the presence of her mother and by her voice, which in turn dissipated Isla’s urge to self-harm. Isla had a realistic comprehension of how much her mother could help; she knew she would still feel upset, but the ‘regulation, nurture and soothing’ reduced the power of the self-harm urge for Isla.

*I think if someone just sat down and spoke to me and took my mind off of it, or did something to distract me, it may make the urges not so prominent in my mind. (Davina)*

*When I feel like self-harming here, because obviously I don’t have the means to, I’ll like go and speak to someone and I’ll go and sit in a communal area with other people and it’s just like at home I’ll just sort of phone up my friends and I’ll have a girly time… (it) will always help me. (Hannah)*

While in an inpatient centre Hannah sought out the company of people within the community, whom she believed understood her and her experiences; she did not have to explain how she was feeling, people ‘got it’. When Hannah was at home she turned to her mother, whom she described as ‘not understanding’ her self-harming, but ‘accepting’ of it and in turn, of Hannah, which she expressed as ‘all she needs’ and to know that ‘someone is there’.

There will be unique factors to every case of self-harm but perhaps the core convergent feature to ‘not self-harming’ was the presence of people, or a person, who can show the individual acceptance, empathy, understanding and unconditional positive regard. These supportive individuals could possibly act as a regulator to the distressing, powerful urges experienced. This was evident in both outpatient relationships and within the inpatient centre. These relationships proved more successful at preventing our participant from self-harming than ‘methods’ suggested by clinicians.

## 4. Discussion

We set out to reveal in-depth reasons why young women engage in NSSI and what helps them to stop. We recruited an all-girl sample for two reasons: due to potential differences in motivations between girls and boys (boys are more likely to be motivated by anger at self or others, while girls are more likely to be motivated by ’self-punishment’ and ‘wanting to communicate distress’ [6,18]) and because IPA requires in-depth exploration of small homogenous sample groups [22]. This means that if we recruited boys as well, we would have needed a much larger sample to be able to establish similarities and differences across genders, which was not feasible in the scope of this project.

Based upon our research questions and grounded in the data, we identified the ‘experience of self-harm’ to encompass mental, emotional, physical and social features.

Self-harm was expressed as an interpersonal means to express and communicate mental distress, to make internal pain visible to self and others, or to quell mental anguish by focusing instead on a physical pain; for example, the pain of cutting and watching the blood flow enabled a participant ‘to feel’—which was preferable to a state of ‘nothingness’. Intrapersonal features were identified in self-punishment and self-loathing.

Self-harm was experienced as compulsive urges, within a ‘vicious cycle’ and as an internal battle between uncontrollable intrusive physical and mental urges, which were sated only by self-harming.

All participants experienced a beneficial reduction in their urges to self-harm in the company of people who understood, tolerated or accepted them. The participants sought the company of family, friends or another, who, by their presence or soothing words, were able to calm the individuals and distract them from their need to self-harm. We suggest that this could be understood within the theoretical context of regulatory interpersonal relationships.

Five themes emerged in our study.

The main finding for engaging in self-harm, consistent with existing literature, is to regulate unpleasant affect [5,6,8,9,10,11,12]. This is not just sad mood, but also anger and numbness. Self-harm leads to a sudden release of those feelings, sometimes by shifting the focus from mental to physical pain [11,33]. Seeing blood also helped participants improve affect, above and beyond the physical pain. Authors of two qualitative studies have demonstrated that seeing blood makes young people who self-harm feel calm [11,33]. Approximately half of a community sample of 64 self-harming young adults reported that it was important to see blood (5) as it relieved tension and led to feelings of calmness. Of note, those for whom seeing blood was important had engaged in more NSSI episodes, possibly making that sub-sample more similar to our clinical sample. These reasons were similar to those stated by participants in this study: to feel calmer, to show they are still alive and to feel “good”. This emphasis on blood may help explain why cutting is the commonest form of self-harm, and why distraction techniques are less effective.

Secondly, NSSI appears similar to substance addiction. Participants described four of the six ICD-10 diagnostic criteria for substance dependence: strong compulsion/craving, lack of control, tolerance and, as revealed in theme 4, persisting with the behavior despite adverse consequences. Indeed, self-harm has been recently been conceptualized as an addictive behavior [34]. Nixon, Cloutier, and Aggarwal [35] found that 41/42 self-harming adolescents demonstrated at least three DSM-IV symptoms of dependence, including tolerance, persisting despite recognizing it as harmful, social problems, and tension recurring if the behavior is stopped. There is strong co-occurrence of both disorders, both longitudinally and cross-sectionally [36,37]. Such overlap may be due to shared genetic and/or environmental risk factors [38,39]. Environmental correlation is more obvious, given the non-specific effects of adversities such as childhood abuse. Genetic correlation may arise through dispositions such as impulsivity or disorders such as borderline personality disorder, both of which increase the risk for NSSI and substance dependence [16,39].

NSSI is often thought of as an impulsive behavior. This is partly because of the societal belief that self-harm is irrational, so people must be doing it without thinking and hence are being impulsive. Support for this idea has come from studies in which young people were shown endorsing cards stating “I did it on impulse without planning” [40] and findings of increased impulsivity as rated by questionnaires [41,42]. However, we have shown that it is sometimes is not impulsive, with young people having the urge for a long time or delaying self-harm until they are alone. Self-harm could, therefore, be also seen as a compulsive behavior, like substance dependence. Compulsivity has been defined as “performance of repetitive and functionally impairing overt or covert behavior without adaptive function, performed in a habitual or stereotyped fashion, either according to rigid rules or as a means of avoiding perceived negative consequences” [43] (p. 2). NSSI is repetitive. People engage in it despite its harmful effects, and it is used to avoid negative affect. All participants in our study engaged in long-term repetitive NSSI. It is possible that self-harming was initially impulsive, but gradually became compulsive, as has been seen in substance dependence [44]. Further studies with people at different stages of NSSI are needed to explore this.

Thus, repetitive NSSI may best be conceptualized as a compulsive disorder, along with addictions, OCD and binge eating disorder [45]. Further studies are needed to test whether there are similar neurocognitive deficits (such as model-free (habit) acquisition) in people with NSSI. If so, pharmacological and psychological treatments for compulsive disorders may be therapeutic for NSSI.

Thirdly, NSSI is used as a survival method. While non-suicidal and suicidal self-harm is sometimes seen as on a continuum of behaviors that should not be separated [46] we have presented evidence that NSSI may be the opposite of suicidality. Young people sometimes self-harm because they want to reduce their suicidal thoughts and live. By reducing intense negative affect, they have learnt that NSSI reduces their suicidality. Some of the evidence for the continuum is based on data from people admitted to hospital for self-harm [46]. These people are likely to be people with severe self-harm, as most self-harm incidents do not lead to admission. A quarter of adolescents who engaged in NSSI had attempted suicide in one large international study [47]. It is important to consider that the young people we see in the community with self-harm may be reducing suicide risk rather than engaging in suicidal behavior. Similar findings have emerged in other qualitative studies [8,11]. We must remember that the underlying negative affect does indicate increased suicide risk and that NSSI is associated with future attempted and completed suicide. However, we should not assume suicidality, but instead, enquire about this sympathetically—the latter is welcomed by patients, the former is not [8].

Some clinicians and families may believe that young people should be stopped from all types of self-harm, if necessary, with intrusive monitoring from parents. Given its potential for reducing suicide risk, we need to think carefully, on a case-by-case basis, whether it is helpful to ask families to try to stop all self-harm in their adolescent. The risks and benefits need to be weighed up carefully.

Providing treatment for the underlying distress and alternative helpful strategies may be more helpful than immediate intrusive attempts to eradicate self-harm.

NSSI was often triggered by negative interpersonal events. This is in keeping with prior qualitative [15] and quantitative [17] findings. Thus, improving relationships, or improving how young people deal with relationships, may help reduce NSSI. Indeed, Interpersonal Psychotherapy-Adolescent-Intensive, a therapy for self-harm/suicidality that specifically deals with relationships, has been shown to reduce suicidality and self-harm in young people [48].

As well as looking at causes for NSSI, we also asked participants about what stops them self-harming. One therapeutic approach is to suggest that young people do other activities that cause pain but less damage, such as flicking an elastic band or holding an ice cube against their wrist. This did not reduce participants’ urge to self-harm. This may be because their tolerance for pain meant they needed a more painful activity (such as self-harm) to cause relief. It may also be because the presence of blood, not just pain, helps to relieve distress. On the other hand, spending time with a supportive family member or friend was beneficial. This has important implications for treatment—it may be more helpful to advise patients who self-harm to seek out and spend time with a positive person, rather than use a mechanical distraction to reduce self-harm. While this needs testing in further research, recent evidence of harm minimization strategies advocated in clinical practice, aimed to reduce or replace self-harm with a ‘safer’ proxy behaviour, such as ‘snapping an elastic band on one’s wrist’, have been shown to be ineffective in real life [29]. In Wadman et al. (2019) [49], some young people questioned the validity of these methods, “that do not work”, and held concerns that their underlying issues, related to self-harm, were not being addressed. Davies et al. (2020) [50] similarly echo the need to adopt a broad repertoire of techniques for managing self-harm because while some young people do experience positive benefits of harm reduction techniques, some describe them as having short-lived effects. Indeed, Birch et al. (2011) [51] explored a positive risk taking approach to self-harm in three women’s in-patient units; arguing that neither a restrictive approach nor a permissive harm minimization approach engendered psychological safety. Instead, they argued that ‘relational security’ can provide psychological security and containment, enabling a reduction in self-harm behaviour. Birch et al. (2011) [51] supported this notion, witnessed in participants being encouraged to communicate feelings of wanting to self-harm, within the context of time and company with staff and fellow patients, thus acknowledging the purpose of their self-harm and enabling the development of secure attachments.

One’s sense of self, and ability to self-regulate, arises from an interpersonal context [52]. Winnicott (1967) espoused that it is in the ‘other’ that the infant finds and internalises his own mind or intentional state, which provides the function of ‘containment’. We suggest that for some individuals, overwhelming urges to self-harm exemplify distressing physiological drivers, which prompt a return to homeostasis, via regulating, albeit dysfunctional, self-harm behaviour. Regulation also involves communicating one’s state to others and bringing the self into existence [52]. Self-harm is seen to fulfil this regulatory and communicative role, enlisting the support of others [5,53,54].

There were similarities and differences with the extant qualitative literature on self-harm. Many studies, like ours, have used female-only participants; a major difference is that we are one of the few studies to use an adolescent rather than an adult sample [13,55]. This is important given that self-harm usually starts in adolescence [47], and only a small proportion of adolescents who self-harm continue to self-harm into adulthood [13]. Thus, our paper may contain a sample closer in time to the start of self-harm (and thus with a better memory of how this started) and a wider range of self-harm severity.

### 4.1. Strengths and Limitations

The main strength of our study is the use of in-depth interviews to explore reasons for self-harming and not self-harming. This allowed us to elicit in-depth motivations and experiences in a way that would be impossible via a tick-box quantitative questionnaire. Qualitative research aims to produce descriptions and interpretations about phenomena under investigation; in particular, IPA recognises that participants’ subjective, perceptual and particular accounts can illuminate convergences and divergences across a single phenomenon. This is both a strength and a criticism of IPA; small samples sizes and the idiographic aspect do not feasibly facilitate generalisations, however commonalities across cases can provide implicit explanations and useful insights, which can have wider implications [56,57].

Our main limitation is the limits of our sample, due to the scope and size of this student project. We needed to focus on adolescent girls (to make sample size manageable) and recruit from clinical services (to facilitate after-care of potentially high-risk adolescents). It is possible that young people with sporadic self-harm, less severe mental health problems and/or who do not want specialist help, have different motivations. This has not been studied in detail to date, and we recommend further research on this, particularly on whether/how motivations change over the course of an individual’s self-harm trajectory, especially in the shift from more sporadic to more recurrent self-harm. It is likely that these results would not be fully generalizable to boys, who have different motivations to self-harm. However, the limits of the sample were put in place to allow more depth, necessary for qualitative research. This can lead to hypothesis generation that can be empirically tested in quantitative studies, such as refinements to current treatment approaches.

### 4.2. Clinical Implications

NSSI is often used to provide relief from intense unpleasant affect. It can sometimes reduce suicidal risk, providing support for the idea that non-suicidal self-injury exists. Therefore, it may be helpful to consider the risks and benefits of self-harm for young people rather than automatically seeing it as something to be eradicated. Further research is needed to test whether external methods of stopping NSSI are helpful or harmful to young people.

When we advise young people who want to stop self-harming, it may be better to advise them to seek out the company of individuals who can help regulate their distress, provide distraction and tolerate their affect, rather than suggesting mechanical methods. We are mindful of the potential for contagion amongst peers, the distress this may cause other young people and of the potential for some family members to be perceived as the ‘source’ of a young person’s distress. Therefore, it is important to carefully consider with the young person who they should approach for support and whether they are suitable, both in terms of their ability to contain affect in a beneficial way and their own vulnerability to feeling distressed if self-harm is discussed with them. Additionally, therapy (such as IPT/IPT-A-IN) that targets interpersonal relationships may help reduce the underlying urge to self-harm.

Repetitive self-harm may be best seen as a compulsive behavior. This needs further testing, which may lead to treatment based on reducing compulsivity.

## Supplementary Materials

The following are available online at https://www.mdpi.com/1660-4601/18/7/3361/s1, The Interview Guide: Understanding Female Adolescent Self-Harm Study Research Interview Schedule and Proposed Interview Questions.
