# Supplementary material for: A Qualitative Study of Understanding Reasons for Self-Harm in Adolescent Girls
Source: Int J Environ Res Public Health. 2021 Mar 24;18(7):3361. doi: 10.3390/ijerph18073361 (PMC8037877; doi:10.3390/ijerph18073361)
Supplement: Supplementary file 1 [file ijerph-18-03361-s001.pdf]

Understanding Female Adolescent Self-Harm Study Research Interview Schedule and Proposed Interview Questions

The proposed research process has a collaborative aspect in that participants are interviewed verbally; we will utilise a semi-structured interview format, using open questions that allow the researcher to be flexible and enable the participants to share as much or as little as the choose. This method affords an already vulnerable participant a measure of power and control in the research relationship. This is particularly relevant to this cohort.

Interviews will last a maximum of one hour. Interviews will be audiotaped, transcribed and anonymised by MM prior to analysis.

The main topics of the interviews will be:

- - Reasons for 1st episode of self-harm
- - Reasons for subsequent episodes of self-harm
- - What stops people self-harming in situations when they would otherwise self-harm
- - Could participants ask for help, and how did people react to the disclosure
- - If participants could choose an ideal way for people to treat them after self-harm or

  disclosing their self-harm, what would they suggest

  This study design has been reviewed by a Patient Participation group, with the assistance of the PPI lead. Members of this group comprised individuals who have lived-experience of self-harm and voluntarily collaborate in the development of research proposals on this topic. This will help to ensure, as far as possible, that those individuals, who have experienced self-harm, can voice their valid opinion on the proposal, the type and tone of questions I plan to ask and on the structure of the interview. This collaborative aspect to the design of the research will help to ensure I have explored the sensitive nature of this research as much as possible with users who have direct experience of the research topic.

Semi-structured Interviews - Potential Interview Questions

Context

MM to set context and explain what will happen in the interview. Participant will be in control of the audio recording button.

Name... "Do you understand that we are here to talk about your experience of self-harm? We are doing this study to learn from people like you, how you experience your self-harm. We would like to understand self-harm a bit better.

I appreciate you being here and participating in this research.

I understand that talking about your experience might be hard for you. You can stop the interview and the recording at any time. And that's OK.
Let me know if you need a break. If you feel that you need to stop the interview and that you cannot carry on, that is also OK. If you do decide to stop, the decision will not affect your usual treatment.

Are you OK to begin?"

Q: Can we start by you telling me a bit about yourself?

- o Age
- o Family / parents / brothers / sisters o School

Q: What about friends? How would you describe your friendships?
Q: Are you okay for me to begin asking you about your experience of self-harm?

- What do you remember about the first time you self harmed?
- How did you feel after the experience?
- Were you able to tell anyone or ask for help?
- How would you describe people's reactions when you told them?

Subsequent episodes of self-harm:
Q: What about other times when you self-harmed, what can you tell me about those?

Q: What would stop you from self-harming, in situations where you may usually self-harm?

Q: If you could choose an ideal way for people to treat you after self-harming, or disclosing your self-harm, what would you suggest?

Protocol to follow if participant becomes distressed:

MM has considered and discussed with her supervisors, the potential risk for a participant to become distressed during the interview or to self-harm following the interview. There could be a potential risk of affect dysregulation, where talking about the behaviour or its meaning could cause emotional distress to the participant. In her training as a counselling psychotherapist, MM has been taught how to recognize signs of distress during a consultation, and how to bring the participant back to a calm state by the end of the interview. If the participant is still upset, MM will ask the participant if they would like her to speak to their friend/family member with them (Community Clinic) or a clinician (Both inpatient and outpatient centres).

The clinician referring the patient will let MM know of the current risk levels of the participant before the research assessment, in particular current suicide risk. This will help MM to ascertain whether any information given in the interview is old (and hence already known about and managed by the clinical team) or new, and hence in need of being passed to the clinical team. Interviews will be carried out in the working day (9-5 Monday to Friday) for outpatients or in the in-patient centre for inpatients so trained clinical staff are available to advise if MM is concerned.

The clinical team already knows of non-suicidal self-injury (which is the reason for referral to the trial). The clinical team may or may not know of suicidal intent, and the participant may reveal that during the interview. If the participant does say they have thoughts of wanting to be dead, or have attempted to take their life in the last month, MM will ask the questions from the risk screen and act as appropriate:

If the participant talks about current thoughts of wanting to be dead during the interview, the following set of questions will be asked:

- Have things felt so bad in the last month that you have thought of trying to take your own life?
- (If no, no more questions)
- Have you actually tried to kill yourself?
- Does anybody else know about this? Do your parents know?
- Do you think you might try to take your life in the next few days?

  If the answers are the same as the patient's clinician already knows, then no further action is needed. If the patient reveals a greater level of risk than is already known about, then following escalation procedure is needed.

Action for Community Patients at Community Mental Health Centre:

- If yes to 'Do you think you might try to take your life in the next few days?' or you have other concerns about safety, speak to Dr Wilkinson (study PI), or the patient's clinician or the on-call clinician at Clinic before the participant goes home
- If yes to 'Have things felt so bad in the last month that you have thought of trying to take your own life?' but no to 'Do you think you might try to take your life in the next few days?' speak to Dr Wilkinson (study PI), or the patient's clinician or the on- call clinician at Clinic within 24 hours for advice on how to act. Tell the participant that you are going to have to speak to a clinician and that they may advise you to tell the patient's parents and GP.
- If the patient says they have had thoughts of wanting to be dead but answer no to 'Have things felt so bad in the last month that you have thought of trying to take your own life?' then the patient's clinician should be informed at the next available opportunity.

Action for In-Patients at the Inpatient Centre

• The participant's named nurse on the current shift, or the nurse in charge, should be informed directly after the interview

The interviews about self-harm may lead to emotional distress for MM, the interviewer. She will discuss any distress with her supervisors. She will increase her weekly private therapy to therapy twice a week.

The most important ethical concern is the safety of the participants and the confidentiality of the information given to the research team by participants. Our ethical responsibility is to maximize patient safety, which means letting other people know if they are at significant risk of suicide, so that risk can be mitigated.

The researcher will speak to Dr Wilkinson, as PI, or the clinical team if concerned about risk to the patient or others. They will advise on how to handle risks and whether to breach confidentiality to parents/carers in line with current Service policies and procedures.
